# Morphological and Cyto-Nuclear Conflicting Signals Across Non-Sister Lineages in Darkling Beetles (Tenebrionidae: *Akis*)

**DOI:** 10.3390/genes17040455

**Published:** 2026-04-14

**Authors:** Pilar Jurado-Angulo, Ernesto Recuero, José L. Ruiz, Mario García-París

**Affiliations:** 1 Department of Biodiversity and Evolutionary Biology, Museo Nacional de Ciencias Naturales (MNCN, CSIC), c/José Gutiérrez Abascal, 2, 28006 Madrid, Spain; pilarjurado@cibio.up.pt (P.J.-A.); ernesto.recuero@urjc.es (E.R.); 2 CIBIO, Centro de Investigação em Biodiversidade e Recursos Genéticos, InBIO Laboratório Associado, BIOPOLIS Program in Genomics, Biodiversity and Land Planning, Campus de Vairão, Universidade do Porto, 4485-661 Vairão, Portugal; 3 Departamento de Biologia, Faculdade de Ciências, Universidade do Porto, 4169-007 Porto, Portugal; 4 Departamento de Biología, Universidad Rey Juan Carlos, c/Tulipán s/n, Móstoles, 28933 Madrid, Spain; 5 Instituto de Estudios Ceutíes, Paseo del Revellín 30, 51001 Ceuta, Spain; euserica@hotmail.com

**Keywords:** marker discordance, Coleoptera, western Palearctic, morphological traits

## Abstract

**Background/Objectives**: Cyto-nuclear discordances, resulting from the independent evolutionary histories of cytoplasmic and nuclear genomes, often obscure phylogenetic inference and species delimitation, particularly at shallow taxonomic levels. In this study, we examine the extent and causes of cyto-nuclear discordances within the darkling beetle tribe Akidini (Coleoptera: Tenebrionidae), focusing on the genera *Akis* Herbst, 1799 and *Morica* Dejean, 1834. **Methods**: Using two molecular markers—nuclear histone 3 (*H3*) and mitochondrial cytochrome *c* oxidase subunit I (*COI*)—and a comprehensive sampling from western Europe and northern Africa, we assess reciprocal monophyly, internal relationships, and phylogenetic incongruence across datasets. **Results**: Discordances between morphological species assignment and mitochondrial topologies may result from retained ancient polymorphisms or historical introgression among closely related species (e.g., *Akis genei* vs. *Akis lusitanica*). However, these causes seem less plausible for explaining discordances between nuclear and mitochondrial markers involving non-closely related species (e.g., *A. discoidea* and *A. granulifera*). The geographic location of the problematic specimens, limited to a narrow marginal contact zone between the two non-sister species, suggests that local hybridisation may occur. **Conclusions**: Our results indicate that cyto-nuclear discordances between mitochondrial and nuclear markers, even across morphologically well-differentiated non-sister lineages, may be more frequent than previously assumed in darkling beetles, highlighting both their evolutionary relevance and the need for caution when relying solely on mitochondrial data for species identification.

## 1. Introduction

The phylogenetic conflict across molecular markers is generally the result of differences in the models of molecular evolution affecting different genes or DNA fragments [1,2,3] or even different sections of a single gene [4]. The problem becomes even more complex when genes from different evolutionary backgrounds, e.g., nuclear vs. cytoplasmic, are compared or analysed together [5,6,7,8]. Organelle (mitochondrion or chloroplast) and nuclear genes not only differ in their model of evolution but also in highly relevant traits such as ploidy, inheritance mode, recombination, and demography [8,9]. These differences often result in conflicting or potentially misleading phylogenetic signals, particularly at shallow evolutionary scales, when markers are compared or analysed together. Evolution of nuclear DNA is also heterogeneous, with substantial differences among markers subject to highly different evolutionary models (e.g., coding, ribosomal, spacers…) [2,10].

Phylogenetic conflict among markers is often assumed to be minimised by combining and analysing together large genome datasets [11,12,13,14], under the expectation that the statistically predominant signal reflects the true phylogenetic relationships of the studied organisms. However, such an approach may not always be reliable: phylogenetic hypotheses can differ according to the datasets included [8,15,16], or may fail to accurately reflect the underlying evolutionary history [17,18,19,20]. As suggested by coalescent theory [21,22,23,24], combining large genomic datasets is likely to provide reliable inferences for deep phylogenetic relationships when taxon sampling is thorough [25,26,27], but it is very unlikely that it works out equally well for more recent, e.g., shallow in time, phylogenetic hypotheses [28,29].

One further, less explored drawback of combining datasets for phylogenetic inference is the potential loss of relevant evolutionary information provided by each individual dataset. The hypothesis derived from loci sharing a common evolutionary mode depicts a “true” phylogeny, i.e., the evolutionary history of the marker analysed for the lineage studied [16,30]. However, such information can be masked or diluted when combined with another “also true” hypothesis, generated either using super-trees or by analysing concatenated datasets that include markers with different evolutionary modes of even different evolutionary or demographic histories [31,32,33]. This loss of information is particularly relevant when the phylogeny includes taxa or lineages that have only recently acquired reproductive incompatibility and may therefore still show evidence of recent past hybridisation or gene introgression [34,35,36], or even phylogenies that include older taxa but with very different demographic histories, resulting in substantially different amount of evolutionary change across them [37]. In these cases, combining datasets will render a single phylogenetic hypothesis that primarily reflects the evolutionary history of the dominant dataset (i.e., the one with the largest proportion of informative characters, irrespective of its informative content), thereby failing to capture the independent information provided by each dataset [32,38].

This problem is particularly relevant when combining mitochondrial and nuclear datasets for phylogenetic reconstruction of shallow-time phylogenies, such as those comprising species within a single tribe, genus, or species–group [7,39]. When combining cytoplasmic and nuclear data that independently produced different topologies, direct information on the processes generating each topology may be lost. This issue has long been discussed in studies on mtDNA evolution, which have shown that the spatial position of secondary contact zones can shift by tens to hundreds of kilometres when inferred from mtDNA compared with nuclear and morphological data [40,41]. Most of these studies have been conducted in well-studied taxa that already have substantial documentation regarding hybridisation or secondary contact zones. In most other groups, the extent and frequency of hybridisation or gene introgression across secondary contact zones is mostly unknown, making it difficult to determine the extent of information lost when combining datasets.

Beetles of the family Tenebrionidae (darkling beetles) are one of these less studied groups in which cyto-nuclear discordances have been already reported without further investigation [42]. Darkling beetles are a main component of the biodiversity of arid and semi-arid regions all over the world. Most species are relatively common and easy to identify at the genus level, but the internal taxonomic structuring within genera is often complex and problematic. In such cases, molecular data are extremely useful for defining evolutionary units and delimiting species hypotheses. However, there is very little information on the extent and frequency of cyto-nuclear discordances across the family, which renders the use of combined datasets not necessarily reliable for certain intra-genus phylogenetic approximations.

In this study, we address this issue by focusing on two of the most conspicuous genera of the Old World Tenebrionidae: *Akis* Herbst, 1799 and *Morica* Dejean, 1834 (tribe Akidini), with two main objectives. First, to assess the reciprocal monophyly and preliminary internal phylogenetic relationships of each genus by analysing two molecular markers, nuclear histone 3 (*H3*) and mitochondrial cytochrome *c* oxidase subunit I (*COI*), in a robust taxon sampling of Akidini from western Europe and Northern Africa. This sampling included 10 species of *Akis* (28% of the world diversity) and three of *Morica* (60% of the world diversity), with most species represented by multiple populations and individuals. Second, to identify discordances between molecular markers (nuclear vs. mitochondrial) and morphological data by contrasting the topologies obtained for each individual marker with the morphological traits of each specimen, and to explore the causes of discordances. We found that phylogenetic discordances in Akidini are likely a consequence of retained ancestral polymorphisms or historical past introgression among recently differentiated taxa or closely related species but may also result from recent processes of hybridisation across secondary contact zones between non-sister species of well-differentiated clades. With this information, we evaluate the utility of analysing independently molecular datasets of diverse nature, even if they are phylogenetically consistent.

## 2. Materials and Methods

### 2.1. Taxon Sampling and DNA Sequencing

Specimens of *Akis* and *Morica* were collected from the Iberian Peninsula, Balearic Islands, and North Africa. The sampling included 10 species of *Akis* (110 specimens) and three species of *Morica* (10 specimens), as well as four specimens of *Leptoderis collaris* (Linnaeus, 1767) (tribe Elenophorini) used as outgroup in the phylogenetic analyses (Table 1).

Specimens were captured by hand, injected with 96% ethanol and preserved in absolute ethanol at −20 °C at the Museo Nacional de Ciencias Naturales (MNCN-CSIC, Madrid). Tissues for DNA extraction were obtained from under the metacoxal plates of the ethanol-preserved individuals.

Subsequently, these tissues were digested and the DNA was purified using the Qiagen DNeasy Blood & Tissue (Qiagen, Hilden, Germany). Partial sequences of the mitochondrial cytochrome *c* oxidase subunit I (*COI*), mitochondrial molecular marker that is informative at both interspecific and intraspecific levels, and the gene histone 3 (*H3*), a nuclear marker with a slower mutation rate than the former, were amplified by polymerase chain reaction (PCR). The set of primers LCO1490 [43]/COI-H [44] and TY-J-1460 [45]/COI-H [44] for the *COI*, and HexAF/HexAR [46] for the *H3* were used. PCR amplifications were performed in a final volume of 25 μl, including 1 μl of template DNA, 0.2 μl of DNA polymerase (Biotools, Madrid, Spain, 5 U/μl), 1 μl of each primer (10 μM), 1 μl of dNTP mix (10 μM), 0.25 μl of MgCl_2_ (50 mM), and 2.5 μl of reaction buffer (Tris–HCl, pH 8.3, Biotools). PCR for *COI* was run with the following conditions: initial denaturation at 94 °C for 5 min, followed by 35 cycles at 94 °C for 30 s, 42–44 °C for 45 s and 72 °C for 1 min, and a final extension step at 72 °C for 5 min. PCR cycling profile for *H3* was: initial denaturation at 94 °C for 5 min, followed by 35 cycles at 94 °C for 40 s, 50 °C for 40 s and 72 °C for 40 s, and a final extension step at 72 °C for 5 min. PCR products were checked in a 1.5% agarose gel and the products of expected length were purified with ethanol–sodium acetate and sequenced in automatic Sanger sequencers by SECUGEN (Madrid, Spain).

Sequences were compiled and revised with the software Sequencer v.5.4.8 (Gene Codes Corporation, Ann Arbor, MI, USA), and aligned manually. Alignments were visually inspected and checked for potential presence of stop codons using Geneious Prime 21.1.1 software. *H3* sequences were assembled as a single consensus sequence per individual from forward and reverse Sanger reads. Heterozygous sites were retained and coded using standard IUPAC ambiguity codes, and no allele phasing was performed.

### 2.2. Phylogenetic Analyses

**Figure 1:**
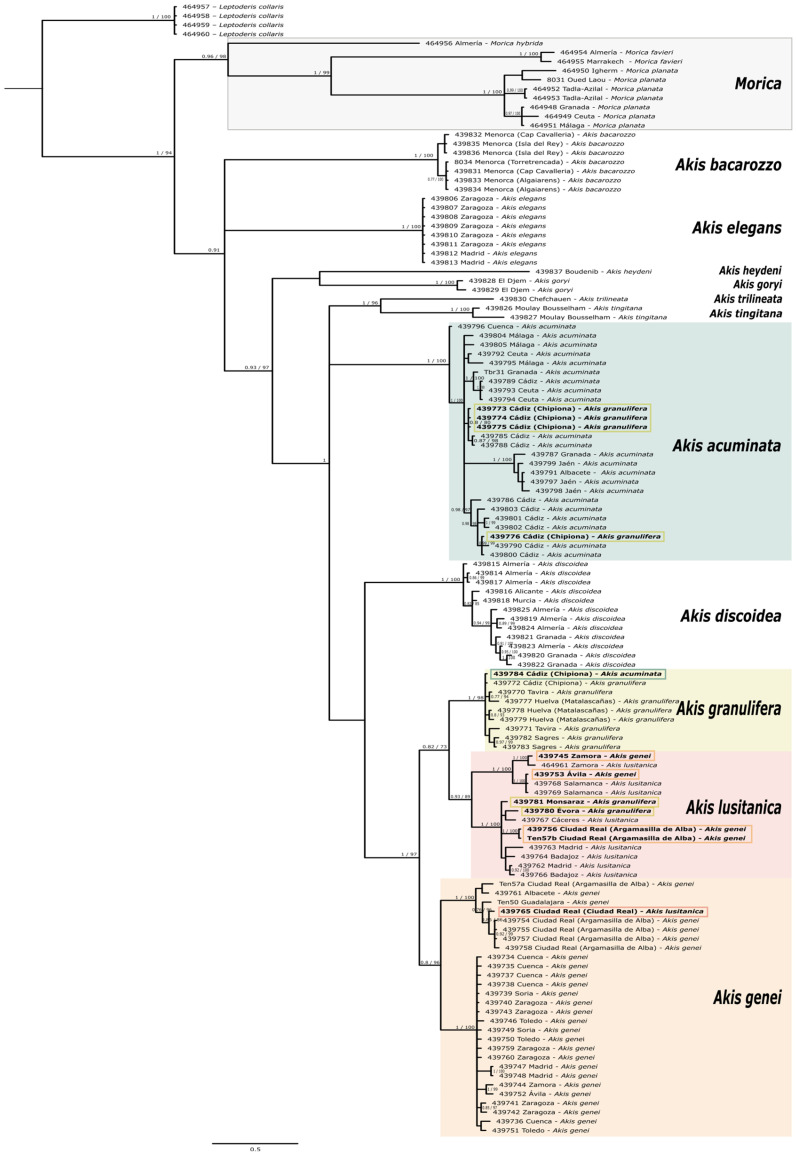
Phylogenetic hypothesis of the analysed representatives of the tribe Akidini, based on mtDNA (*COI*) sequences. The same topology was recovered using Bayesian inference and maximum-likelihood approaches. Node support (PP/UFBS) is indicated when equal or higher than 0.75/75. Names shown on the right margin indicate the mitochondrial clades of the genus or species included in the analyses, while terminal branch labels indicate the code, locality, and morphological identification of each sample. Specimens whose morphology does not correspond to their mtDNA clade are highlighted with a box coloured according to the mitochondrial clade of the morphologically identified species (i.e., green for *A. acuminata*, yellow for *A. granulifera*, pink for *A. lusitanica*, and orange for *A. genei*).

**Figure 2:**
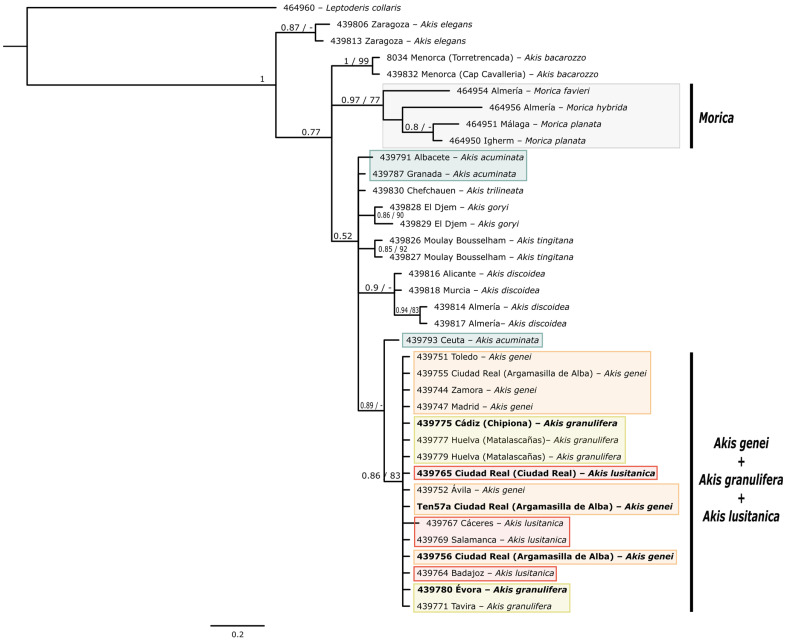
Phylogenetic hypothesis of the analysed representatives of the tribe Akidini, based on nDNA (*H3*). Node support (PP/UFBS) is indicated when equal or higher than 0.75/75. Terminal branches indicate the code, locality, and morphological identification of each sample. Samples in bold indicate specimens showing discrepancies between mtDNA and morphology; coloured boxes group species following the colour code used in the other figures.

**Figure 3:**
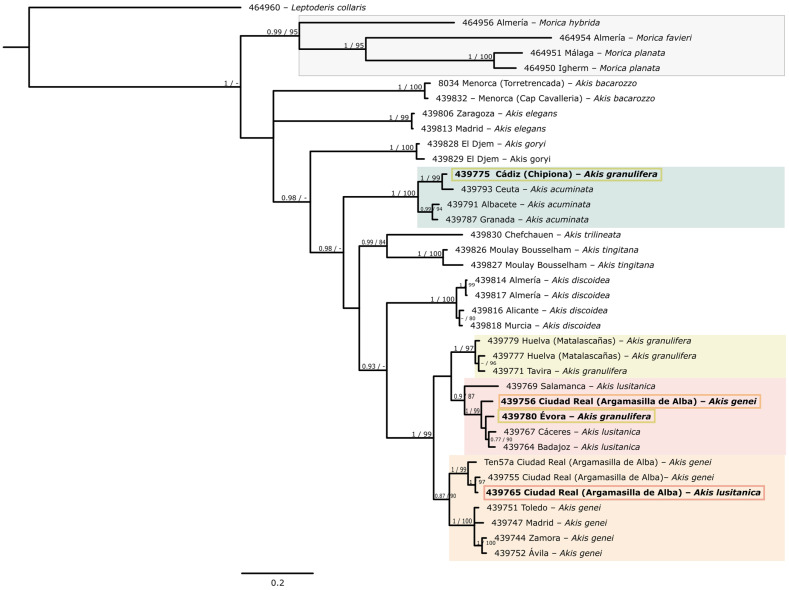
Phylogenetic hypothesis of the analysed representatives of the tribe Akidini, based on the combined dataset (*COI* and *H3*). Node support (PP/UFBS) is indicated when equal or higher than 0.75/75. Terminal branches indicate the code, locality, and morphological identification of each sample. Specimens whose morphology does not correspond to their mtDNA clade are highlighted with a box coloured according to the mitochondrial clade of the morphologically identified species (i.e., green for *A. acuminata*, yellow for *A. granulifera*, pink for *A. lusitanica*, and orange for *A. genei*).

Three different datasets were constructed for the phylogenetic analyses: one for each sequenced gene (Figure 1 and Figure 2) and a third comprising both genes concatenated (Figure 3). First, the best-fit substitution models for each gene were determined using JModelTest ver. 2.1.10 [47]. These models were subsequently applied in the Bayesian analyses, both for the individual gene assessments and the combined analysis. In the latter, two partitions were defined based on the genes, considering the different models applicable to each gene.

We assessed substitution saturation for *COI* using the entropy-based Xia test implemented in DAMBE (DAMBE7) [48], evaluating all sites and codon partitions (1st + 2nd vs. 3rd positions) using 60 replicates; Iss was compared to the critical Iss.c under both symmetrical and asymmetrical topologies.

The analyses were conducted in MrBayes ver. 3.2.6 [49] and comprised two simultaneous runs of 10 million generations, with trees sampled every 1000 generations. Convergence of the Monte Carlo Markov chains (MCMC) were verified using Tracer v.1.7 [50], by examining trace plots and Effective Sample Size (ESS). A 20% “burn-in” was applied to remove the initially topologies. The final results were summarised in a Maximum Clade Credibility (MCC) tree using TreeAnnotator v.1.8.4 [51], and posterior probability values (PP) were considered as an estimate of the stability.

We also analysed the three datasets using a maximum-likelihood phylogenetic approach in IQ-TREE v2.4 [52]. Node support was assessed with 1000 ultrafast bootstrap (UFBS) replicates, and the best-fitting substitution model was selected automatically for each locus using the “-m AUTO” option. Trees were rooted using the outgroup *Leptoderis collaris.*

To establish a temporal framework for the cladogenesis events observed in our analyses, we employed BEAST version 1.10.4 [53]. We analysed the *COI* database under a GTR+I+G nucleotide substitution model, and a Birth and Death tree prior. Because no reliable fossil calibrations exist for this lineage, we used a secondary, rate-based calibration derived from a well-characterised biogeographic calibration event and estimated over six different Tenebrionidae genera [54]. We used an uncorrelated lognormal relaxed clock, placing a normal prior on the parameter ucld.mean (mean = 0.0169 substitutions per lineage per Myr; SD = 0.0019), following the estimates reported for tenebrionid beetles [54]. This rate prior assumes that the mitochondrial *COI* rate is broadly transferable across Tenebrionidae, as commonly adopted in studies lacking fossil constraints. The analyses ran for 20 million generations, sampling trees and other parameters every 2000 generations. The stability and convergence of the results were verified using Tracer v.1.7 [50], with an optimal burn-in of 25%. We used TreeAnnotator v1.10.4 to generate a MCC tree, with posterior probabilities to assess node support.

### 2.3. Species Delimitation Analyses

We performed distance-based, single-locus species delimitation analyses separately for the genera *Akis* and *Morica* using ASAP (Assemble Species by Automatic Partitioning) [55]. ASAP analyses were run via the Spart Explorer platform (https://spartexplorer.mnhn.fr) [56] using our *COI* dataset, corresponding with the barcoding region typically used in Metazoa [57]. We used simple uncorrected p-distances and set the split-group probability threshold to 0.001. ASAP scores and panmixia probabilities (*p*-value) were used to select the best-supported species partitions.

Following the recommendations of the ASAP framework [55], we treated ASAP outputs as preliminary species hypotheses within an integrative taxonomy workflow. To reduce potential over-splitting inherent to single-locus, distance-based approaches, we cross-validated ASAP partitions against independent morphological and geographic evidence before drawing final taxonomic conclusions (Table S1).

### 2.4. Morphological Study

Morphological study was based on the original species descriptions and on the diagnostic characters proposed by zur Strassen [58] and Ferrer et al. [59]. The main diagnostic morphological characters of each species included in this study are summarised in Table S2. Specimens showing discordance between morphological identification and mtDNA were photographed in order to illustrate the observed morphological variability. Photographs were taken using a digital camera Nikon (Nikon Corporation, Tokyo, Japan) and a lens Nikon AF-S VR Micro-Nikkor 105 mm f/2.8 G IF-ED, using the software Helicon Remote ver. 3.9.11 and Helicon Focus ver. 7.6.4.

## 3. Results

### 3.1. Species Delimitation

Among species of the genus *Akis*, ASAP analysis resulted in two similarly supported partitions (ASAP score = 4, *p*-value = 2.355289 × 10^−1^/6.467066 × 10^−1^ for 13/14 species partition, respectively), suggesting the existence of 13 to 14 species hypotheses. These values are slightly higher than our morphological hypotheses, by splitting, as potential different species, clades morphologically defined as *A. genei*, *A. lusitanica* (13 species partition) and also *A. acuminata* (14 species partition). Considering all available evidence (molecular, morphological, and geographic distribution), we consider that ASAP results are slightly overestimating species diversity in those three lineages, as frequently observed for single-locus species delimitation methods [55], and that an integrative taxonomy species delimitation includes 11 species of *Akis* within our sampling. For the genus *Morica*, we obtained a single, best supported partition (ASAP score = 1, *p*-value = 2.215569 × 10^−1^) including three species, perfectly matching morphological and geographic evidence (Table S1).

### 3.2. Interspecific Relationships

Xia’s test showed no evidence of saturation when considering all sites (e.g., for the largest subset, NumOTU = 32: Iss = 0.153, Iss.cAsym = 0.393, t = 14.533, df = 662, *p* < 0.001). For 3rd codon positions, Iss slightly exceed the critical value (NumOTU = 32: Iss = 0.406, Iss.cAsym = 0.366), but the associated test was not statistically significant (t = 1.264, df = 220, *p* = 0.208). This result suggests faster synonymous evolution at third positions but does not provide significant statistical support for substitution saturation under Xia’s criterion. Results were consistent across alternative NumOTU subsets and under the symmetrical topology.

The Bayesian analysis of the partial sequences of *COI* mtDNA gene and the two combined genes (*COI* and nuclear *H3*) confirmed the monophyly of *Akis* and *Morica*, respectively (Figure 1 and Figure 3). The analysis of the *H3* gene on its own (Figure 2), due to its limited variability, does not provide a statistically well-resolved phylogenetic reconstruction. While it does recover a monophyletic clade for the genus *Morica* (PP = 0.97; UFBS = 77), the relationships within the genus *Akis* are less clear, with the position of the lineages corresponding to *A. elegans* Charpentier, 1825 and *A. bacarozzo* Schrank von Paula, 1786 being particularly contentious. Within the genus *Morica*, our results suggest a basal position for the Iberian species *M. hybrida* Charpentier, 1825, while *M. favieri* Lucas, 1859 appears more closely related to *Morica planata* Fabricius, 1801 (Figure 1 and Figure 3).

**Figure 4:**
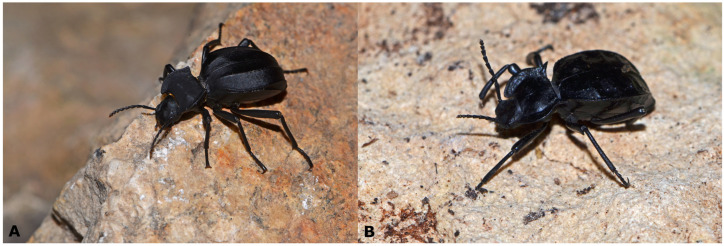
Specimens of *Akis elegans* and *Akis bacarozzo*. (**A**) Female specimen of *A. elegans* from El Viso de San Juan (Toledo, Spain). (**B**) Male specimen of *A. bacarozzo* from Menorca (Spain).

Some relationships within *Akis* are more difficult to resolve confidently due to the low phylogenetic resolution of both markers when analysed independently (Figure 1 and Figure 2). However, the existence of three main, distinct lineages seems evident: two of them each corresponding with the species *A. elegans* and *A. bacarozzo* (Figure 4), and a third one including the remaining species. Within the latter group, *A. goryi* Solier, 1836 and *A. heydeni* Haag-Rutenberg, 1876 emerge as sister species, while the position of *A. acuminata* Fabricius, 1787 remains ambiguous. In the concatenated analysis, *A. acuminata* appears as the basal lineage of the entire group (PP = 0.98, UFBS = 74; Figure 3), but in the analysis of *COI*, it forms a polytomy with the clade that groups *A. tingitana* Lucas, 1859 and *A. trilineata* Herbst, 1799, and the clade consisting of *A. discoidea* Quensel, 1806, the sister species to the *A. granulifera* Sahlberg, 1823 group (which includes *A. granulifera*, *A. genei* Solier, 1836, and *A. lusitanica* Solier, 1836) (Figure 1). Finally, the *A. granulifera* species group consistently forms a monophyletic clade. In both the *COI* and concatenated analyses (Figure 1 and Figure 3), *A. lusitanica* and *A. granulifera* are more closely related to each other than to *A. genei*. However, in the *H3* analysis (Figure 2), it is not possible to establish relationships between these species, as all sequenced *H3* alleles from these three species are almost identical and nested in a single clade with no structure.

**Figure 5:**
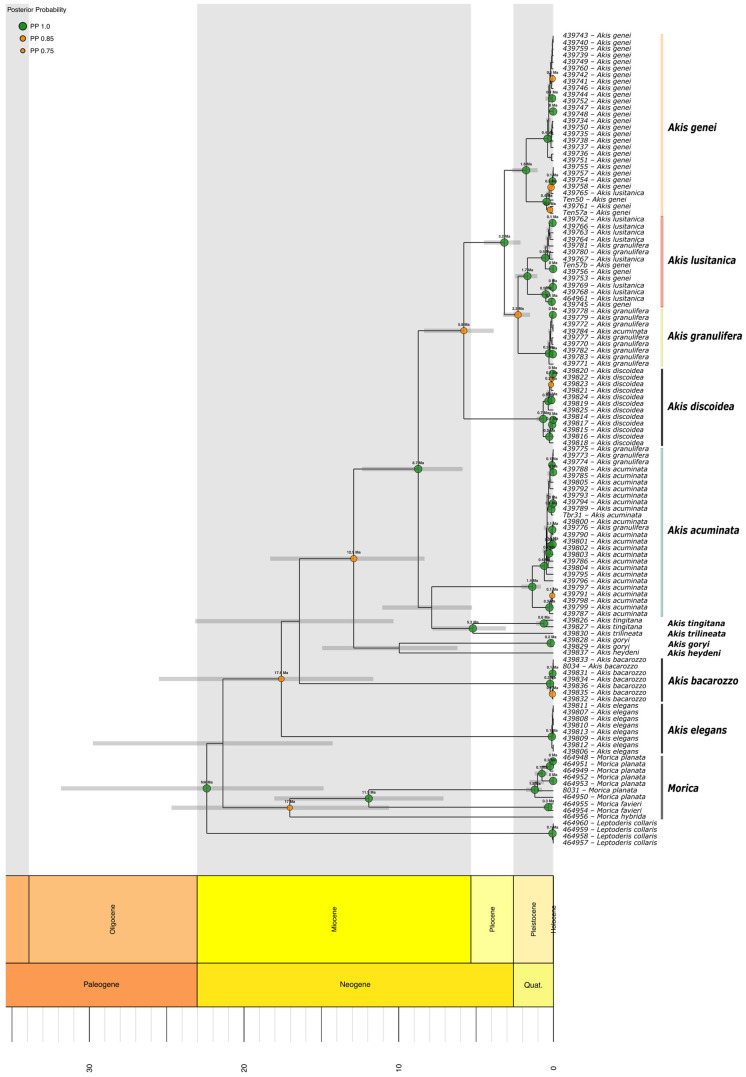
Chronogram showing mean divergence ages of the analysed lineages of Akidini estimated in BEAST from the *COI* dataset. Posterior probability (PP) is shown when higher than 0.75. Grey horizontal bars at nodes indicate 95% highest posterior density (HPD) intervals. Coloured vertical bars on the right group mitochondrial clades and follow the species colour coding used in the other figures, regardless of instances of mito-nuclear discordance.

The phylogenetic analysis of the *COI* sequences using a relaxed molecular clock suggests relatively old divergence times (Figure 5). The Iberian group comprising the taxa *Akis discoidea*, *A. granulifera*, *A. lusitanica*, and *A. genei* shows an estimated age of their common ancestor between 3.9 and 8.4 million years ago (Ma), with a mean estimated value of 5.79 Ma, with an ancestor for the three latter species estimated in the late Pliocene, around 3.17 (2.1–4.5) Ma. The Iberian–African group, which includes the aforementioned species plus *A. acuminata*, *A. tingitana*, and *A. trilineata*, would have an estimated age between 5.9 and 12.4 Ma, with a mean estimated value of 8.74 Ma. The age of the clade including this group and the African species represented in our analysis by *A. goryi* and *A. heydeni* is estimated between 8.3 and 18.3 Ma, with a mean estimated value of 12.91 Ma. The common ancestor for all *Akis* species included in the analysis occurred around 17.6 (11.6–25.5) Ma. The estimated divergence times among species of *Morica* are even older than those among species of the genus *Akis*, although the base of the clade (the split of *M. hybrida* from the rest) has a similar age estimate of 17.04 (10.6–24.7) Ma. The time to the most recent common ancestor of *M. planata* and *M. favieri* was estimated at 11.93 (7.1–18) Ma. Several of the studied species show an intraspecific genetic diversity originating during the Pleistocene, over 1 Ma, including *Akis acuminata* (1.37, 0.8–2.1 Ma), *A. genei* (1.77, 1–2.7 Ma), *A. lusitanica* (1.68, 1–2.5 Ma), and *Morica planata* (1.2, 0.7–1.8 Ma).

### 3.3. Intraspecific Relationships

At the intraspecific level, *Akis genei* presents two divergent clades (Figure 1) with almost parapatric geographic distribution. The northern clade is broadly distributed north and south to the Iberian Central System, in the Spanish provinces of Soria, Zaragoza, Madrid, Toledo, and Cuenca. The southern group has a more restricted distribution, with populations located in the provinces of Ciudad Real, Albacete, and Guadalajara. Based on the distribution of these two groups, it is likely that a broad contact zone between the two lineages exists in La Mancha.

The lineage of *A. lusitanica* comprises two distinct subclades that are clearly separated by the Central System. A northern subclade is present in Salamanca, Ávila, and Zamora, while a southern subclade extends across Extremadura and central Portugal (Figure 1). Haplotypes assignable to *A. lusitanica* have even been found as far north as Valdefinjas (Zamora). A population of possibly introduced origin has been detected inside the city of Madrid.

Some specimens morphologically assignable to *A. genei* from Ávila and Zamora, present mitochondrial haplotypes corresponding to *A. lusitanica* (Figure 1). Although *A. genei* and *A. lusitanica* exhibit mostly separate geographic distributions, mitochondrial haplotypes of *A. lusitanica* appear in some specimens from populations morphologically assignable to *A. genei* (in Zamora and Ávila), while mitochondrial haplotypes clustering with *A. genei* are displayed in populations assignable to *A. lusitanica* (Ciudad Real).

**Figure 6:**
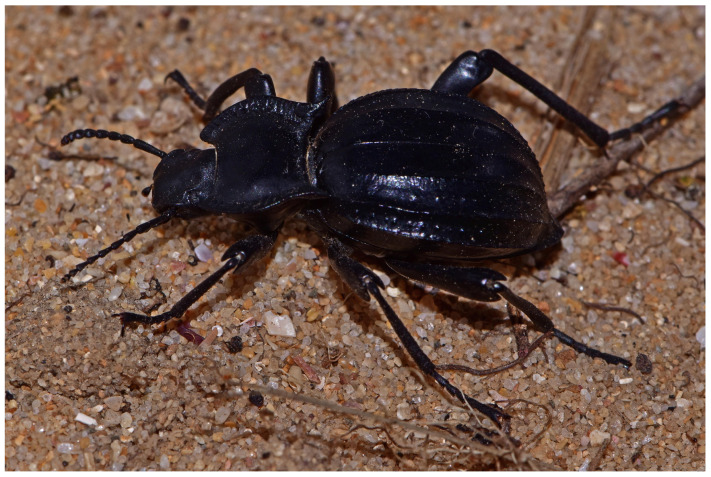
Male specimen with typical morphology of *A. granulifera* from Sagres (Faro, Portugal).

**Figure 7:**
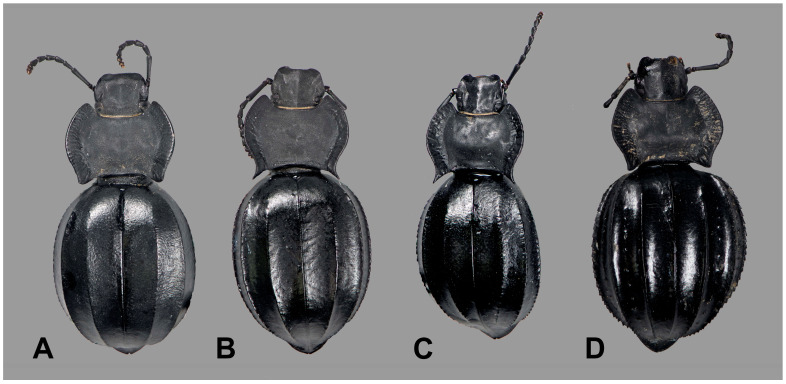
Dorsal view of specimens of the *Akis granulifera* and *Akis lusitanica* mtDNA clades. (**A**) Female specimen with typical morphology of *A. lusitanica* included in the mtDNA clade of *A. lusitanica*, from Calera de León (Badajoz, Spain) (MNCN_Ent 439764) with moderately shiny elytra, matte pronotum, well developed but not very sharp elytral costae, and only the external serrate; (**B**) Male specimen from Mitra (Évora, Portugal) (MNCN_Ent 439780) with morphology somewhat transitional between *A. lusitanica* and *A. granulifera*, including very shiny elytra, sharp costae serrated, included in the mtDNA clade of *A. lusitanica*; (**C**) Male specimen from Tavira (Faro, Portugal) with all morphological traits and geographic provenance, as considered by zur Strassen [58] and Ferrer et al. [59], of *Akis bayardi*, included in the mtDNA clade of *A. granulifera* (MNCN_Ent 439771); (**D**) Female specimen from Matalascañas (Huelva) corresponding morphologically and geographically to *Akis ilonka*, included in the mtDNA clade of *A. granulifera* (MNCN_Ent 439779). See also Figure 6 showing a typical specimen of *Akis granulifera* from Sagres (Faro, Portugal).

**Figure 8:**
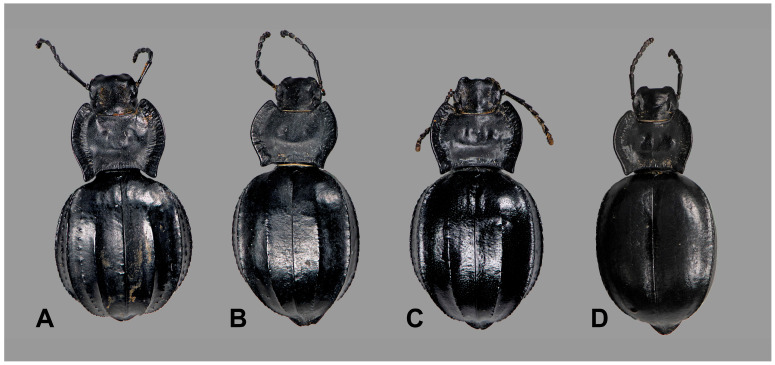
Specimens from the contact zone between *A. acuminata* and *A. granulifera* in Chipiona (Cádiz, Spain). (**A**) Specimen with typical morphology of *A. granulifera* (MNCN_Ent 439774) from the Cádiz area, but having mtDNA corresponding to *A. acuminata*; (**B**) Specimen with almost typical morphology of *A. granulifera* (MNCN_Ent 439773) with a mtDNA haplotype corresponding to *A. acuminata*; note that the internal costae are less prominent and intercostal tubercles reduced; (**C**) Another specimen with less typical morphology of *A. granulifera* (MNCN_Ent 439776) with mtDNA haplotype of *A. acuminata*; note that the intercostal tubercles are absent; (**D**) Typical *Akis acuminata* (MNCN_Ent 439786) from Puerto Real (Cádiz, Spain).

Mitochondrial haplotypes of specimens showing the typical morphology of *A. granulifera* (Figure 6), *A. ilonka*, and *A. bayardi* (Figure 7C,D) are grouped into a single clade, which is the sister group to *A. lusitanica* (Figure 1 and Figure 7A). This lineage includes typical *A. granulifera* specimens (from Faro district, Portugal) (Figure 6), individuals corresponding to the morphology of *A. bayardi* (from Tavira, Portugal) (Figure 7C), and those of *A. ilonka* (from Matalascañas, Spain) (Figure 7D). In the locality of Chipiona (Cádiz, Spain), we also found specimens exhibiting typical *A. granulifera* morphology but with *A. acuminata* mtDNA, and vice versa (Figure 8).

Within the *A. acuminata* clade, genetic differentiation is relatively low (Figure 1). The most distinct haplotypes, though lacking a clear geographical structure, correspond to specimens from the Iberian Peninsula’s interior: Tarancón (Cuenca), Villarrobledo (Albacete), Pegalajar (Jaén), and Darro (Granada). The specimens from Albacete and Cuenca notably extend the species’ known distribution inland. The absence of genetic differentiation between populations located on either side of the Strait of Gibraltar is particularly remarkable (Figure 1). Some haplotypes are shared between populations from Ceuta (North Africa) and Cádiz (Southern Europe), while others are very similar between Ceuta and Málaga (Fuengirola). The *A. acuminata* clade also includes two haplotypes found in four specimens that are morphologically assignable to *A. granulifera* from Chipiona (Cádiz) (Figure 1 and Figure 8). Therefore, in Chipiona, there are specimens with *A. granulifera* morphology and *A. acuminata* mtDNA haplotypes, and also specimens with *A. acuminata* morphology and *A. granulifera* mtDNA. At Chipiona, we also found some specimens with peculiar morphological traits, which could be considered intermediate characteristics between the two species (Figure 8B,C). Some of these individuals exhibited the general appearance of *A. acuminata* but had an additional elytral ridge, matching the morphology described for *A. acuminata dorsigera* [60,61].

The *Akis* specimens studied on the island of Menorca correspond to the morphology of *A. bacarozzo* and its synonym, *A. tuberculata*. For the genetic analyses, specimens with the morphology of *A. bacarozzo* (from Torretrencada Cap Cavalleria, and Algaiarens) (Figure 4B) and with the morphology of *A. tuberculata* (from Cap Cavalleria and Algaiarens) were examined. The results (Figure 1) indicate that, regardless of their morphology or origin, all the specimens cluster into a single clade comprising only two haplotypes that differ by a single base pair.

The *A. discoidea* clade shows relatively high variability compared to other species of the genus (Figure 1), with all studied specimens showing the typical morphology of *A. discoidea*, and no clear geographical structure.

For the *A. elegans* clade, a noteworthy finding is a poor mitochondrial diversity, with only a single haplotype detected across the five populations studied from Madrid and Zaragoza.

In *Morica planata*, we found five different *COI* haplotypes. The largest diversity is concentrated in Morocco, with four highly divergent haplotypes (Figure 1). In contrast, only one haplotype has been found in the Iberian Peninsula, which is more closely related to the one found in Ceuta than to the others in Morocco. The two *M. favieri* specimens studied, one from southeastern Iberia and another from near Marrakech, exhibit two *COI* haplotypes that are only slightly divergent (Figure 1).

## 4. Discussion

### 4.1. Phylogenetic Relationships and Morphological Diversity Within Akis and Morica

The concatenated analyses resulted in a phylogenetic hypothesis with a topology very similar to that obtained from the mtDNA dataset alone (Figure 1 and Figure 3), depicting *Morica* and *Akis* as reciprocally monophyletic and providing resolution within the *Akis granulifera* species group. Given the similarity between the combined and mtDNA trees, it is evident that nuclear information is largely overshadowed in the concatenated dataset, even though posterior probability values for some nodes increased upon concatenation. A plausible explanation for this is that the number of informative characters differs substantially between the mtDNA dataset (34.8%) and the nuclear dataset (8.5%), thereby obscuring the less informative dataset.

The combined and mtDNA dataset broadly supports the morphological species hypotheses traditionally used to define species within *Akis*, with all taxa recovered as monophyletic (except for a few terminal branches which are discussed below). Although the nuclear dataset is less informative overall and depicts clades with low support, it provides some relevant insights for species–group delimitation. For instance, all Iberian taxa related to *A. granulifera* (including *A. lusitanica* and *A. genei*) form a tight clade of poorly differentiated *H3* sequences (PP = 0.86) (Figure 2). Estimates of the time to the most recent common ancestor, based on our *COI* sequences, indicate a Pliocene origin for this clade, with pre-Pleistocene speciation events (Figure 5). Considering these ages, the substitution rate of the studied *H3* fragment appears too low to generate meaningful differentiation, at least within clades 2–3 million years old. As with any mtDNA-based dating that relies on a single substitution rate, some uncertainty is expected due to possible rate variation among lineages and divergence depths. Absolute ages should therefore be interpreted with caution, although the relative divergence patterns are robust. In fact, the topologies obtained from the different datasets are not in conflict but are rather unresolved. Thus, the main discrepancies observed are not in the main phylogenetic hypotheses but are related to individual assignments to different clades when using mtDNA or nDNA data.

Relationships within the genera *Akis* and *Morica* have been little discussed. Zur Strassen [58] and Español [60] considered *A. granulifera*, *A. lusitanica* and *A. genei* to be closely related, which is fully supported by our mtDNA and nuclear topologies (Figure 1 and Figure 2). They even proposed *A. granulifera* and *A. lusitanica* as subspecies of the same species. Indeed, we have identified populations in southern Portugal attributable to *A. lusitanica* but showing morphological traits reminiscent of *A. granulifera* (Figure 7), including the loss and fading of elytral tubercles and modifications in the shape of the elytral costae. This suggests the potential existence of a broad contact zone between *A. lusitanica* and *A. granulifera*, which warrants further in-depth study. Based on detectable levels of genetic exchange between *A. granulifera* and *A. lusitanica* in this region, the taxonomic status of these two species could be reconsidered, potentially supporting the taxonomic hypotheses proposed by zur Strassen [58] and Español [60]. However, both *A. lusitanica* and *A. granulifera* retain relatively stable morphological characteristics across their extensive geographical distributions. Furthermore, both groups exhibit a degree of differentiation comparable to that observed between *A. genei* and *A. granulifera*. Thus, in line with previous authors [59,62], we propose to treat *A. lusitanica* as an independent evolutionary unit and maintain its species-level taxonomic status while more detailed data become available.

The clade containing haplotypes from specimens with typical *A. granulifera* morphology (from Chipiona, Spain) also includes haplotypes from specimens exhibiting *A. bayardi* morphology (from Tavira, Portugal) (Figure 7C) [59], as well as haplotypes from all specimens with *A. ilonka* morphology (Matalascañas and Chipiona, Spain) (Figure 7D). The genetic differentiation observed among haplotypes within this clade is minimal, suggesting that the morphological variation should either be considered as individual variability or as reflecting the presence of local ecotypes. Specifically, the *A. ilonka* population does not differ genetically from other *A. granulifera* populations. Consequently, the hypotheses treating *A. ilonka* as a distinct species [59,63] are rejected. According to the principle of priority (Article 23 of the International Code of Zoological Nomenclature) [64], the name *Akis granulifera* Sahlberg, 1823, takes nomenclatural precedence over *Akis bayardi* Solier, 1836, and *Akis ilonka* zur Strassen, 1957, which should be treated as synonyms of *A. granulifera.*

Regarding the morphological differentiation observed within *Akis discoidea*, we lack adequate molecular data for the morphotypes named as *A. hispanica* Solier, 1836 and *A. salzei* Solier, 1836, as our analyses only included specimens with typical *A. discoidea* morphology. However, based on the morphological descriptions of *A. hispanica* and *A. salzei*, their traits appear to align with the levels of phenotypic plasticity observed in southeastern *A. granulifera* populations. Viñolas & Cartagena [62] and Español [60] previously considered *A. salzei* and *A. hispanica* as recurring individual variations in *A. discoidea*. Ferrer et al. [59], however, treated *A. salzei* and *A. hispanica* as separate species, whereas Iwan et al. [65] synonymised *A. salzei* with *A. hispanica*. Given that specimens of *A. hispanica* and *A. salzei* recorded to date are intermixed with typical *A. discoidea* specimens [59], we consider it plausible that both, *A. hispanica* and *A. salzei*, represent patterns of individual variability within *A. discoidea*. Nevertheless, testing this hypothesis would require the inclusion of these phenotypes in future molecular analyses.

Soldati [66] synonymised *A. dorsigera* Reitter, 1904, with *A. acuminata* (Fabricius, 1787), considering the previous elevation to species status by Ferrer et al. [59] to have been based on “irrelevant (and incorrectly illustrated) character states.” Our molecular analyses included a specimen exhibiting typical *A. dorsigera* morphology, characterised by the general appearance of *A. acuminata* but with an additional elytral ridge [60,61]. This specimen, collected in Chipiona (Cádiz), presented mtDNA assignable to *A. granulifera*. Therefore, we support Soldati’s [66] hypothesis. It would be worthwhile to investigate the genetic situation in other localities where this unusual morphology has been recorded (in Córdoba and Cádiz provinces and southern Portugal) [59,61] to identify the processes underlying this morphological pattern (Figure 8).

Ferrer et al. [59] elevated *Akis tuberculata* Kraatz, 1865 to species rank, which Leo & Fanchello [67] later synonymised with *Akis bacarozzo* Schrank von Paula, 1786. Specimens from Menorca morphologically assignable to *A. tuberculata* were genetically identical to those morphologically assignable to *A. bacarozzo* based on the molecular markers studied (Figure 1). Thus, we concur with the taxonomic proposals considering both morphological types to represent a single taxon, with *A. bacarozzo* taking nomenclatural priority [60,62,63,67].

### 4.2. Biogeography

Based on our results, several distinct groups within the Akidini tribe can be identified in the western Mediterranean, potentially shaped by the formation of the Betic Riffean Massif. Iberian populations of *M. planata* are recently separated from the Moroccan stock and therefore unrelated to the closure of the Strait of Gibraltar (5.3 million years ago) [68]. In contrast, the divergence of *M. hybrida* with respect to *M. planata* appears to be considerably older. Our estimates of the clade’s age (Figure 5) are inconsistent with the hypothesis that ancestral *M. hybrida* colonised the Iberian Peninsula during the Messinian Salinity Crisis and was later isolated following the reopening of the Strait of Gibraltar. This scenario would only be plausible if these organisms exhibited significantly higher mutation rates than those typically observed in other insects. An alternative explanation could be that *M. hybrida* became isolated in the Betic Riffean Massif, which formed during the Early to Middle Miocene [69].

For *Akis*, we identified a distinct Iberian–African group comprising four clades (Figure 1): two African species (*A. tingitana* and *A. trilineata*), one species found on both sides of the Strait of Gibraltar (*A. acuminata*), and a fourth clade comprising four Iberian endemics (*A. discoidea*, *A. genei*, *A. lusitanica*, and *A. granulifera*). Although we were unable to fully resolve the basal relationships among these clades, our dating places the origin of this Iberian–African group between the Middle and Late Miocene (Figure 5). This suggests that the formation of the various clades could be linked to isolation events following the reopening of the Betic Strait, which separated the Iberian Peninsula from the Betic-Rif Massif. This pattern is similar to the speciation scenarios proposed by Martínez-Solano et al. [70] for *Alytes* (Anura).

Our phylogeographic analyses further demonstrate that the movement of terrestrial species across the Strait of Gibraltar, traditionally considered a strong barrier, has occurred more frequently than previously thought [71,72,73,74,75]. In this group, dispersal has taken place both northward from Africa to Europe and southward from Europe to Africa (Figure 1). In the case of *A. acuminata*, our data clearly indicate that dispersal occurred from the Iberian Peninsula to North Africa, similar to the pattern observed in *Pleurodeles waltl* Michahelles, 1830 [76,77]. The entire intraspecific diversity of *A. acuminata* is concentrated in the Iberian Peninsula, and there is notable genetic similarity between populations from Ceuta in northern Africa and Cádiz in southern Spain (Figure 1). Since *A. acuminata* lacks the ability to cross large bodies of water like the Strait of Gibraltar on its own, its colonisation likely occurred passively, perhaps via the transport of adult beetles or larvae by rafts of vegetation. The species’ frequent presence in coastal habitats near the sea supports this possibility. However, given its pronounced anthropophilic tendencies, it is also plausible that human activity facilitated its dispersal, as previously suggested by Ruiz & Ávila [78]. This hypothesis is further supported not only by the genetic similarity with Iberian specimens but also by the species’ restricted distribution in North Africa, being limited to areas around Tangier [79] and Ceuta [78], where it is associated with suburban environments subject to significant human impact. The case of *Morica planata* follows a similar pattern to *A. acuminata*, although our data suggest that dispersal in this species occurred in the opposite direction, from south to north (Figure 1), as observed in *Hyla meridionalis* Boettger, 1874 [80] and in various species of darkling beetles of the genus *Pimelia* [42,75]. The analysed samples of *M. planata* show considerable differentiation among African populations, with four highly divergent haplotypes identified in Northern Africa (Figure 1). In contrast, only a single haplotype was found in the Iberian Peninsula, which is closely related to the haplotype found in Ceuta. Genetically, these results suggest that the Iberian populations originated relatively recently and have not yet had time to accumulate significant mutations in their DNA. Regarding the mechanisms of dispersal, the same hypotheses proposed for *A. acuminata* may be applicable, as the two species are frequently found in sympatry. Moreover, the low mitochondrial differentiation observed between the two *M. favieri* specimens studied (Figure 1), one from southeastern Iberia and another from the Marrakech area, also suggests recent colonisation events in southeastern Iberia. The movement of species of *Akis* across the Mediterranean is also evident in multiple directions, with *A. acuminata* and *A. bacarozzo* found in the Balearic Islands [60], and *A. trilineata* documented in Barcelona and Almería [62]. In all these cases of introduction, the role of maritime ports such as Ceuta and Tangier in Africa, or Málaga and Almería in Europe, stands out as potential facilitators of species transport. These colonisation events may go completely unnoticed when populations are widespread on both sides of the Strait, highlighting the importance of phylogeographic studies for their detection.

### 4.3. The Problems of Morphological Variability in Tenebrionid Taxonomy

A major challenge for species definition in Tenebrionidae is that the main morphological taxonomic characters useful for defining species within a clade are often inconsistent or even misleading when applied to closely related clades, making taxonomic decisions impossible or even serendipitous. In these cases, molecular data are extremely useful for defining evolutionary units.

The most commonly used characters for distinguishing species within the tribe Akidini are based on external morphology. Specifically, in the taxonomy of the tribe, the most significant characteristic is the arrangement and structure of the ridges, ribs, and rows of tubercles running longitudinally along the elytra [58,59,60,62,67,81]. These elytral traits are also the most contentious in the identification of *Akis* species, as many species exhibit substantial morphological variability, which has occasionally led to the description of varieties, subspecies, or species of questionable validity. Ferrer et al. [59] provide detailed illustrations of the arrangement and structure of the elytral ridges of all Iberian *Akis* taxa, making it unnecessary to present the data again. Morphologically, the characters outlined by Ferrer et al. [59] accurately distinguish all the examined specimens in this work. However, the core issue, as is common in typological taxonomy, lies in determining whether the observed morphological differences correspond to species-level differences, locally adapted population sets exhibiting differential expression of high phenotypic plasticity, or merely individual variations occasionally arising within a population.

In some cases, the presence of microgranulation on the elytral surface has proven useful and serves as an indisputable diagnostic character for identifying taxa such as *A. genei* and *A. lusitanica*, which frequently coexist and display highly similar external morphologies [59,60]. *Akis lusitanica* and *A. genei* are phylogenetically close species, but their divergence is ancient (Figure 5), and the level of mitochondrial DNA sequence divergence is substantial. Both species occur in sympatry across a broad region of the western plateau, with no evidence of ongoing hybridisation between them, although the observed discordances in geographically scattered specimens showing mtDNA haplotypes that do not match their morphological assignment (e.g., specimens with morphology of A. lusitanica presenting *COI* haplotypes assignable to *A. genei*, and vice versa) suggest the possible existence of either retention of ancestral polymorphisms or local gene introgression (Figure 1). Given the broad sympatric range and the low levels of introgression, it is indisputable that these two lineages represent independent species according to both evolutionary [82] and biological criteria [83]. Our results do not support previous doubts regarding the diagnostic value of microgranulation [81]. Male genitalia can define groups at generic and suprageneric levels [84], however genital morphology is not commonly used for species-level taxonomy of Pimeliinae, as the degree of differentiation between closely related taxa is minimal and intrapopulational variability is so high that potential differences between closely related species are obscured [59]. The female reproductive system seems to follow similar patterns, exhibiting marked differentiation at deep nodes but high intraspecific variability [85,86].

### 4.4. Cyto-Nuclear Discordances and Hybridization

There are some isolated cases of mismatch between the mtDNA phylogenetic hypothesis, the nuclear topology, and the morphological data (Figure 1, Figure 7 and Figure 8). The first case involves four specimens clearly identifiable morphologically as *A. granulifera* and assigned to the *A. granulifera*–*A. lusitanica*–*A. genei* nuclear *H3* clade that fall within the mitochondrial clade of *A. acuminata* (Figure 1 and Figure 8). Conversely, one specimen morphologically assignable to *A. acuminata* and included as such in the nuclear *H3* clade, presents mtDNA of *A. granulifera*.

The *A. granulifera* and *A. acuminata* clades are highly differentiated, not sister to each other, and have been separated since the Miocene (5.9–12.4 Ma) (Figure 5). Both species are well characterised morphologically and are unmistakably diagnosable (Figure 8). All the specimens involved in this situation came from the same area, a small stretch of back-dunes near the inner edge of the beach, flanked by avenues and streets within the village of Chipiona (Cádiz, Spain). Chipiona is almost located at the westernmost edge of the geographic range of *A. acuminata*, and also quite into the easternmost limit for *A. granulifera* [58,59,62,81,87], but the population is predominantly composed of *A. granulifera*, with *A. acuminata* being relatively scarce. It is thus quite possible that contact between these two species is relatively uncommon. Sympatry between these two species has only previously been reported in one locality: “El Conquero”, Huelva [87]. The observed pattern of mtDNA introgression is thus likely the result of occasional local hybridisation following recent secondary contact between these two otherwise unrelated species. It is very likely that the introgression in this case remained confined to the areas where the two species cohabit.

In the absence of rapidly evolving nuclear markers, testing this hypothesis could be achieved by identifying morphologically singular specimens that, although clearly assignable to one species, exhibit some characters typical of the other, indicating potential nuclear gene exchange. Among the seven specimens studied from Chipiona, five showed evidence of mtDNA introgression, but only one displayed divergent morphology. This specimen probably represents a F1 hybrid, and since no other morphological evidence has been observed, we infer that hybridisation is locally restricted and unlikely to be persistent over time.

The second set of mismatches between morphology and mtDNA occurs between the closely related, but non-sister, *A. genei* and *A. lusitanica*. In this case, some specimens morphologically assigned to *A. genei* present mtDNA corresponding to *A. lusitanica* (Zamora, Ávila, Ciudad Real), whereas some specimens morphologically assigned to *A. lusitanica* present mtDNA corresponding to *A. genei* (Ciudad Real). Despite exhibiting highly similar external morphology, both species can be clearly distinguished by their elytral tegument patterns. These mismatched specimens were collected in the southern and northwestern regions of the Sistema Central, respectively. These areas may provide extensive contact zones between *A. genei* and *A. lusitanica*. However, since the haplotypes found are not identical to those currently present in the corresponding species (Figure 1), it is also possible that these cases reflect the retention of ancestral polymorphisms, whereby certain populations have not lost ancient haplotypes inherited from their common ancestors, resulting in incomplete lineage sorting [88]. This process may have been facilitated if *A. lusitanica* exhibited particularly large effective population sizes in these regions [89]. Alternatively, these instances could represent mitochondrial introgression derived from past hybridisation events in past contact zones, allowing the persistence of *A. lusitanica* haplotypes within *A. genei* populations, or vice versa. In this context, ongoing hybridisation need not be persistent, which would account for the absence of specimens with intermediate morphological characteristics or even for the lack of current sympatry. Both phenomena might occur simultaneously, potentially suggesting that *A. lusitanica* is expanding into areas previously inhabited solely by *A. genei*, leading to local displacement. As *A. genei* shifts eastward, traces of introgression may remain. In this regard, it is noteworthy that we have found *A. lusitanica* in Madrid, an area where *A. genei* would traditionally be expected; however, this population may instead result from accidental introduction through soil transport for landscaping, as has already been documented in other cases [90]. Nevertheless, none of these hypotheses challenges the treatment of *A. lusitanica* and *A. genei* as independent taxonomic entities. Even if the observed patterns reflect hybridisation, the levels of introgression are so limited relative to the overall distribution range of both taxa that do not undermine the existence of reproductive isolation between them. Furthermore, the restricted geographical extent of the areas of cyto-nuclear discordances and the substantial divergence observed between *A. lusitanica* and *A. genei* provide additional support for this interpretation.

Our results suggest that cyto-nuclear discordances across morphologically differentiated non-sister clades may be more frequent in darkling beetles than generally assumed (see also Mas-Peinado et al. [42]). This situation provides an opportunity to study the origin and consequences of introgression in organisms that rely primarily on postzygotic mechanisms for reproductive isolation [91,92]. It also indicates that mitochondrial barcoding may not always constitute a reliable tool for species identification in certain groups of arthropods, as it has been observed in other organisms [93,94].

## Figures and Tables

**Table 1:** Specimens used for DNA analyses, with their corresponding specimen codes, localities, and GenBank accession numbers.

Specimen Code	Morphology-Based Species	Locality	*COI*	*H3*
MNCN_Ent 439734	*Akis genei*	Spain: Cuenca: Segóbriga	PZ044878	
MNCN_Ent 439735	*Akis genei*	Spain: Cuenca: Segóbriga	PZ044879	
MNCN_Ent 439736	*Akis genei*	Spain: Cuenca: Segóbriga	PZ044880	
MNCN_Ent 439737	*Akis genei*	Spain: Cuenca: Segóbriga	PZ044881	
MNCN_Ent 439738	*Akis genei*	Spain: Cuenca: Segóbriga	PZ044882	
MNCN_Ent 439739	*Akis genei*	Spain: Soria: Monteagudo de las Vicarías	PZ044885	
MNCN_Ent 439740	*Akis genei*	Spain: Zaragoza: Farlete	PZ044889	
MNCN_Ent 439741	*Akis genei*	Spain: Zaragoza: Farlete	PZ044890	
MNCN_Ent 439742	*Akis genei*	Spain: Zaragoza: 3 km NW Farlete	PZ044891	
MNCN_Ent 439743	*Akis genei*	Spain: Zaragoza: 3 km NW Farlete	PZ044892	
MNCN_Ent 439744	*Akis genei*	Spain: Zamora: Valdefinjas	PZ044896	PZ049398
MNCN_Ent 439745	*Akis genei* (mtDNA *A. lusitanica*)	Spain: Zamora: Valdefinjas	PZ044897	
MNCN_Ent 439746	*Akis genei*	Spain: Toledo: Toledo, Puente de San Martín	PZ044900	
MNCN_Ent 439747	*Akis genei*	Spain: Madrid: Valdaracete	PZ044901	PZ049399
MNCN_Ent 439748	*Akis genei*	Spain: Madrid: Valdaracete	PZ044902	
MNCN_Ent 439749	*Akis genei*	Spain: Soria: Medinaceli	PZ044923	
MNCN_Ent 439750	*Akis genei*	Spain: Toledo: Consuegra	PZ044924	
MNCN_Ent 439751	*Akis genei*	Spain: Toledo: Sierra del Romeral, Villacañas	PZ044934	PZ049395
MNCN_Ent 439752	*Akis genei*	Spain: Ávila: Castro de las Cogotas	PZ044943	PZ049407
MNCN_Ent 439753	*Akis genei* (mtDNA *A. lusitanica*)	Spain: Ávila: Ávila	PZ044945	
MNCN_Ent 439754	*Akis genei*	Spain: Ciudad Real: Argamasilla de Alba	PZ044949	
MNCN_Ent 439755	*Akis genei*	Spain: Ciudad Real: Argamasilla de Alba	PZ044950	PZ049397
MNCN_Ent 439756	*Akis genei* (mtDNA *A. lusitanica*)	Spain: Ciudad Real: Argamasilla de Alba	PZ044951	PZ049412
MNCN_Ent 439757	*Akis genei*	Spain: Ciudad Real: Argamasilla de Alba	PZ044952	
MNCN_Ent 439758	*Akis genei*	Spain: Ciudad Real: Argamasilla de Alba	PZ044953	
MNCN_Ent 439759	*Akis genei*	Spain: Zaragoza: Fuendetodos	PZ044960	
MNCN_Ent 439760	*Akis genei*	Spain: Zaragoza: Fuendetodos	PZ044961	
MNCN_Ent 439761	*Akis genei*	Spain: Albacete: El Bonillo	PZ044983	
Ten50	*Akis genei*	Spain: Guadalajara: Illana-Estremera	PZ044964	
Ten57a	*Akis genei*	Spain: Ciudad Real: Argamasilla de Alba	PZ044965	PZ049409
Ten57b	*Akis genei* (mtDNA *lusitanica*)	Spain: Ciudad Real: Argamasilla de Alba	PZ044966	
MNCN_Ent 439762	*Akis lusitanica*	Spain: Madrid: Madrid, Calle Catalina Suárez	PZ044883	
MNCN_Ent 439763	*Akis lusitanica*	Spain: Madrid: Madrid, Calle Catalina Suárez	PZ044884	
MNCN_Ent 439764	*Akis lusitanica*	Spain: Badajoz: Calera de León	PZ044917	PZ049413
MNCN_Ent 439765	*Akis lusitanica* (mtDNA *genei*)	Spain: Ciudad Real: 4 km N Ciudad Real	PZ044922	PZ049405
MNCN_Ent 439766	*Akis lusitanica*	Spain: Badajoz: Ctra. EX325 Km 6–7 Valdebótoa, N from Badajoz	PZ044925	
MNCN_Ent 439767	*Akis lusitanica*	Spain: Cáceres: Trujillo	PZ044946	PZ049410
MNCN_Ent 439768	*Akis lusitanica*	Spain: Salamanca: Puente del Congosto	PZ044947	
MNCN_Ent 439769	*Akis lusitanica*	Spain: Salamanca: Puente del Congosto	PZ044948	PZ049411
MNCN_Ent 464961	*Akis lusitanica*	Spain: Zamora: Malillos	PZ044982	
MNCN_Ent 439770	*Akis granulifera*	Portugal: Algarve: Tavira, Forte do Rato	PZ044898	
MNCN_Ent 439771	*Akis granulifera*	Portugal: Algarve: Tavira, Forte do Rato	PZ044899	PZ049424
MNCN_Ent 439772	*Akis granulifera*	Spain: Cádiz: Chipiona	PZ044905	
MNCN_Ent 439773	*Akis granulifera* (mtDNA *A. acuminata*)	Spain: Cádiz: Chipiona	PZ044906	
MNCN_Ent 439774	*Akis granulifera* (mtDNA *A. acuminata*)	Spain: Cádiz: Chipiona	PZ044907	
MNCN_Ent 439775	*Akis granulifera* (mtDNA *A. acuminata*)	Spain: Cádiz: Chipiona	PZ044908	PZ049400
MNCN_Ent 439776	*Akis granulifera* (mtDNA *A. acuminata*)	Spain: Cádiz: Chipiona	PZ044909	
MNCN_Ent 439777	*Akis granulifera*	Spain: Huelva: Matalascañas	PZ044910	PZ049401
MNCN_Ent 439778	*Akis granulifera*	Spain: Huelva: Matalascañas	PZ044911	
MNCN_Ent 439779	*Akis granulifera*	Spain: Huelva: Matalascañas	PZ044912	PZ049402
MNCN_Ent 439780	*Akis granulifera* (mtDNA *A. lusitanica*)	Portugal: Alto Alentejo: Mitra, Évora	PZ044926	PZ049414
MNCN_Ent 439781	*Akis granulifera* (mtDNA *A. lusitanica*)	Portugal: Alto Alentejo: São Pedro de Corval, Monsaraz	PZ044927	
MNCN_Ent 439782	*Akis granulifera*	Portugal: Algarve: Fortaleza de Sagres	PZ044977	
MNCN_Ent 439783	*Akis granulifera*	Portugal: Algarve: Fortaleza de Sagres	PZ044978	
MNCN_Ent 439784	*Akis acuminata* (mtDNA *A. granulifera*)	Spain: Cádiz: Chipiona	PZ044903	
MNCN_Ent 439785	*Akis acuminata*	Spain: Cádiz: Chipiona	PZ044904	
MNCN_Ent 439786	*Akis acuminata*	Spain: Cádiz: Puerto Real	PZ044928	
MNCN_Ent 439787	*Akis acuminata*	Spain: Granada: Darro	PZ044930	PZ049408
MNCN_Ent 439788	*Akis acuminata*	Spain: Cádiz: Estella del Marqués	PZ044931	
MNCN_Ent 439789	*Akis acuminata*	Spain: Cádiz: Estella del Marqués	PZ044932	
MNCN_Ent 439790	*Akis acuminata*	Spain: Cádiz: Estella del Marqués	PZ044933	
MNCN_Ent 439791	*Akis acuminata*	Spain: Albacete: Villarrobledo	PZ044937	PZ049406
MNCN_Ent 439792	*Akis acuminata*	Spain: Ceuta: Avenida Madrid	PZ044938	
MNCN_Ent 439793	*Akis acuminata*	Spain: Ceuta: Avenida Madrid	PZ044939	PZ049396
MNCN_Ent 439794	*Akis acuminata*	Spain: Ceuta: Avenida Madrid	PZ044940	
MNCN_Ent 439795	*Akis acuminata*	Spain: Málaga: Fuengirola	PZ044942	
MNCN_Ent 439796	*Akis acuminata*	Spain: Cuenca: Tarancón	PZ044954	
MNCN_Ent 439797	*Akis acuminata*	Spain: Jaén: Pegalajar VG4376	PZ044979	
MNCN_Ent 439798	*Akis acuminata*	Spain: Jaén: Pegalajar VG4376	PZ044980	
MNCN_Ent 439799	*Akis acuminata*	Spain: Jaén: Pegalajar VG4376	PZ044981	
MNCN_Ent 439800	*Akis acuminata*	Spain: Cádiz: Medina Sidonia TF3938	PZ044992	
MNCN_Ent 439801	*Akis acuminata*	Spain: Cádiz: Medina Sidonia TF3938	PZ044993	
MNCN_Ent 439802	*Akis acuminata*	Spain: Cádiz: Medina Sidonia TF3938	PZ044994	
MNCN_Ent 439803	*Akis acuminata*	Spain: Cádiz: Medina Sidonia TF3938	PZ044995	
MNCN_Ent 439804	*Akis acuminata*	Spain: Málaga: Fuente de Piedra	PZ044996	
MNCN_Ent 439805	*Akis acuminata*	Spain: Málaga: Fuente de Piedra	PZ044997	
Tbr31	*Akis acuminata*	Spain: Granada: El Albaicín	PZ044963	
MNCN_Ent 439806	*Akis elegans*	Spain: Zaragoza: Pozuel de Ariza	PZ044886	PZ049393
MNCN_Ent 439807	*Akis elegans*	Spain: Zaragoza: Mequinenza	PZ044887	
MNCN_Ent 439808	*Akis elegans*	Spain: Zaragoza: 5 km al N de Maella	PZ044888	
MNCN_Ent 439809	*Akis elegans*	Spain: Zaragoza: Farlete	PZ044893	
MNCN_Ent 439810	*Akis elegans*	Spain: Zaragoza: Farlete	PZ044894	
MNCN_Ent 439811	*Akis elegans*	Spain: Zaragoza: 3 km NW Farlete	PZ044895	
MNCN_Ent 439812	*Akis elegans*	Spain: Madrid: Fuente El Saz de Jarama	PZ044919	
MNCN_Ent 439813	*Akis elegans*	Spain: Madrid: Fuente El Saz de Jarama	PZ044920	PZ049394
MNCN_Ent 439814	*Akis discoidea*	Spain: Almería: Pulpí	PZ044913	PZ049403
MNCN_Ent 439815	*Akis discoidea*	Spain: Almería: Pulpí	PZ044914	
MNCN_Ent 439816	*Akis discoidea*	Spain: Alicante: Denia	PZ044918	PZ049404
MNCN_Ent 439817	*Akis discoidea*	Spain: Almería: San Juan de Terreros	PZ044921	PZ049425
MNCN_Ent 439818	*Akis discoidea*	Spain: Murcia: Jumilla	PZ044929	PZ049423
MNCN_Ent 439819	*Akis discoidea*	Spain: Almería: El Alquián (Cabo de Gata)	PZ044944	
MNCN_Ent 439820	*Akis discoidea*	Spain: Granada: 6.5 km S–SW Charches	PZ044985	
MNCN_Ent 439821	*Akis discoidea*	Spain: Granada: 6.5 km S–SW Charches	PZ044984	
MNCN_Ent 439822	*Akis discoidea*	Spain: Granada: 6.5 km S–SW Charches	PZ044986	
MNCN_Ent 439823	*Akis discoidea*	Spain: Almería: Laujar de Andarax	PZ044987	
MNCN_Ent 439824	*Akis discoidea*	Spain: Almería: Laujar de Andarax	PZ044988	
MNCN_Ent 439825	*Akis discoidea*	Spain: Almería: Laujar de Andarax	PZ044989	
MNCN_Ent 439826	*Akis tingitana*	Morocco: Moulay Bousselham	PZ044915	PZ049418
MNCN_Ent 439827	*Akis tingitana*	Morocco: Moulay Bousselham	PZ044916	PZ049419
MNCN_Ent 439828	*Akis goryi*	Tunisia: El Djem	PZ044935	PZ049415
MNCN_Ent 439829	*Akis goryi*	Tunisia: El Djem	PZ044936	PZ049416
MNCN_Ent 439830	*Akis trilineata*	Morocco: Garganta Oued Laou, N Chefchauen	PZ044941	PZ049417
AKI8034	*Akis bacarozzo*	Spain: Menorca: Torretrencada	PZ044955	PZ049391
MNCN_Ent 439831	*Akis bacarozzo*	Spain: Menorca: Cala Torta, Cap Cavalleria	PZ044956	
MNCN_Ent 439832	*Akis bacarozzo*	Spain: Menorca: Cala Torta, Cap Cavalleria	PZ044957	PZ049392
MNCN_Ent 439833	*Akis bacarozzo*	Spain: Menorca: Algaiarens	PZ044958	
MNCN_Ent 439834	*Akis bacarozzo*	Spain: Menorca: Algaiarens	PZ044959	
MNCN_Ent 439835	*Akis bacarozzo*	Spain: Menorca: Isla del Rey, Mahón	PZ044990	
MNCN_Ent 439836	*Akis bacarozzo*	Spain: Menorca: Isla del Rey, Mahón	PZ044991	
MNCN_Ent 439837	*Akis heydeni*	Morocco: Boudenib: Kef Aziza	PZ044962	
MNCN_Ent 464948	*Morica planata*	Spain: Granada: Ventas de Zafarraya	PZ044967	
MNCN_Ent 464949	*Morica planata*	Spain: Ceuta: Camino de La Lastra	PZ044968	
MNCN_Ent 464950	*Morica planata*	Morocco: 20 km E Igherm	PZ044971	PZ049422
MNCN_Ent 464951	*Morica planata*	Spain: Málaga: Villanueva del Trabuco	PZ044972	PZ049421
AKI8031	*Morica planata*	Morocco: Oued Laou, Talambote	PZ044973	
MNCN_Ent 464952	*Morica planata*	Morocco: Tadla-Azilal: Kasba Tadla	PZ044974	
MNCN_Ent 464953	*Morica planata*	Morocco: Tadla-Azilal: Kasba Tadla	PZ044975	
MNCN_Ent 464954	*Morica favieri*	Spain: Almería: 8 km E Tabernas	PZ044969	PZ049420
MNCN_Ent 464955	*Morica favieri*	Morocco: Marrakech	PZ044976	
MNCN_Ent 464956	*Morica hybrida*	Spain: Almería: Los Atochares, Níjar	PZ044970	PZ049390
MNCN_Ent 464957	*Leptoderis collaris*	Spain: Madrid: 5 km NE Molino de Aldehuela	PZ044998	
MNCN_Ent 464958	*Leptoderis collaris*	Spain: Madrid: 5 km NE Molino de Aldehuela	PZ044999	
MNCN_Ent 464959	*Leptoderis collaris*	Spain: Zaragoza: Farlete	PZ045000	
MNCN_Ent 464960	*Leptoderis collaris*	Spain: Zaragoza: Mequinenza	PZ045001	PZ049389

## Data Availability

The genetic data supporting the findings of this study are freely available in GenBank (PZ044878-PZ045001, PZ049389-PZ049425). The sequence alignments are available in the Zenodo repository (DOI: 10.5281/zenodo.19040712).

## Supplementary Materials

The following supporting information can be downloaded at: https://www.mdpi.com/article/10.3390/genes17040455/s1, Table S1. Correspondence between ASAP partitions inferred from *COI* sequences and the final species hypotheses adopted in this study. Morphological identification and geographic area were used to evaluate and, when necessary, merge or retain ASAP units within an integrative taxonomic framework; Table S2. Main morphological diagnostic traits of the species of *Morica* and *Akis*. Sources: [58,59,60,62,81,95,96,97], and pers. obs. (authors).
