# Supplementary material for: Morphological and Cyto-Nuclear Conflicting Signals Across Non-Sister Lineages in Darkling Beetles (Tenebrionidae: Akis)
Source: Genes (Basel). 2026 Apr 14;17(4):455. doi: 10.3390/genes17040455 (PMC13116412; doi:10.3390/genes17040455)
Supplement: Supplementary file 1 [file genes-17-00455-s001.zip › Table S1.pdf]

Table S1. Correspondence between ASAP partitions inferred from COI sequences and the final species hypotheses adopted in this study. Morphological identification and geographic area were used to evaluate and, when necessary, merge or retain ASAP units within an integrative taxonomic framework.

Voucher	ASAP Partition	Morphological ID	Geographic area	Final Species ID
MNCN_Ent 439734	A1	*Akis genei*	Central Iberia	*Akis genei*
MNCN_Ent 439739	A1	*Akis genei*	Central Iberia	*Akis genei*
MNCN_Ent 439740	A1	*Akis genei*	NE Iberia	*Akis genei*
MNCN_Ent 439743	A1	*Akis genei*	NE Iberia	*Akis genei*
MNCN_Ent 439749	A1	*Akis genei*	Central Iberia	*Akis genei*
MNCN_Ent 439759	A1	*Akis genei*	NE Iberia	*Akis genei*
MNCN_Ent 439760	A1	*Akis genei*	NE Iberia	*Akis genei*
MNCN_Ent 439735	A1	*Akis genei*	Central Iberia	*Akis genei*
MNCN_Ent 439737	A1	*Akis genei*	Central Iberia	*Akis genei*
MNCN_Ent 439738	A1	*Akis genei*	Central Iberia	*Akis genei*
MNCN_Ent 439750	A1	*Akis genei*	Central Iberia	*Akis genei*
MNCN_Ent 439741	A1	*Akis genei*	NE Iberia	*Akis genei*
MNCN_Ent 439746	A1	*Akis genei*	Central Iberia	*Akis genei*
MNCN_Ent 439751	A1	*Akis genei*	Central Iberia	*Akis genei*
MNCN_Ent 439742	A1	*Akis genei*	NE Iberia	*Akis genei*
MNCN_Ent 439752	A1	*Akis genei*	Central Iberia	*Akis genei*
MNCN_Ent 439736	A1	*Akis genei*	Central Iberia	*Akis genei*
MNCN_Ent 439744	A1	*Akis genei*	Central Iberia	*Akis genei*
MNCN_Ent 439747	A1	*Akis genei*	Central Iberia	*Akis genei*
MNCN_Ent 439748	A1	*Akis genei*	Central Iberia	*Akis genei*
MNCN_Ent 439765	A2	*Akis lusitanica*	Central Iberia	*Akis lusitanica*
MNCN_Ent 439754	A2	*Akis genei*	Central Iberia	*Akis genei*
MNCN_Ent 439755	A2	*Akis genei*	Central Iberia	*Akis genei*
MNCN_Ent 439757	A2	*Akis genei*	Central Iberia	*Akis genei*
MNCN_Ent 439758	A2	*Akis genei*	Central Iberia	*Akis genei*
Ten50	A2	*Akis genei*	Central Iberia	*Akis genei*
Ten57a	A2	*Akis genei*	Central Iberia	*Akis genei*
MNCN_Ent 439761	A2	*Akis genei*	Central Iberia	*Akis genei*
MNCN_Ent 439762	A3	*Akis lusitanica*	Central Iberia	*Akis lusitanica*
MNCN_Ent 439766	A3	*Akis lusitanica*	SW Iberia	*Akis lusitanica*
MNCN_Ent 439764	A3	*Akis lusitanica*	SW Iberia	*Akis lusitanica*
MNCN_Ent 439781	A3	*Akis granulifera*	SW Iberia	*Akis granulifera*
MNCN_Ent 439767	A3	*Akis lusitanica*	Central Iberia	*Akis lusitanica*
MNCN_Ent 439763	A3	*Akis lusitanica*	Central Iberia	*Akis lusitanica*
MNCN_Ent 439780	A3	*Akis granulifera*	SE Iberia	*Akis granulifera*
MNCN_Ent 439756	A3	*Akis genei*	Central Iberia	*Akis genei*
Ten57b	A3	*Akis genei*	Central Iberia	*Akis genei*
MNCN_Ent 439745	A5	*Akis genei*	Central Iberia	*Akis genei*
MNCN_Ent 464961	A5	*Akis lusitanica*	Central Iberia	*Akis lusitanica*
MNCN_Ent 439753	A5	*Akis genei*	Central Iberia	*Akis genei*
MNCN_Ent 439768	A5	*Akis lusitanica*	Central Iberia	*Akis lusitanica*
MNCN_Ent 439769	A5	*Akis lusitanica*	Central Iberia	*Akis lusitanica*

MNCN_Ent 439806	A4	*Akis elegans*	E Iberia	*Akis elegans*
MNCN_Ent 439807	A4	*Akis elegans*	E Iberia	*Akis elegans*
MNCN_Ent 439808	A4	*Akis elegans*	E Iberia	*Akis elegans*
MNCN_Ent 439809	A4	*Akis elegans*	E Iberia	*Akis elegans*
MNCN_Ent 439810	A4	*Akis elegans*	E Iberia	*Akis elegans*
MNCN_Ent 439811	A4	*Akis elegans*	E Iberia	*Akis elegans*
MNCN_Ent 439812	A4	*Akis elegans*	E Iberia	*Akis elegans*
MNCN_Ent 439813	A4	*Akis elegans*	E Iberia	*Akis elegans*
MNCN_Ent 439770	A6	*Akis granulifera*	SE Iberia	*Akis granulifera*
MNCN_Ent 439784	A6	*Akis acuminata*	S Iberia	*Akis acuminata*
MNCN_Ent 439772	A6	*Akis granulifera*	S Iberia	*Akis granulifera*
MNCN_Ent 439778	A6	*Akis granulifera*	S Iberia	*Akis granulifera*
MNCN_Ent 439779	A6	*Akis granulifera*	S Iberia	*Akis granulifera*
MNCN_Ent 439782	A6	*Akis granulifera*	SE Iberia	*Akis granulifera*
MNCN_Ent 439783	A6	*Akis granulifera*	SE Iberia	*Akis granulifera*
MNCN_Ent 439777	A6	*Akis granulifera*	S Iberia	*Akis granulifera*
MNCN_Ent 439771	A6	*Akis granulifera*	SE Iberia	*Akis granulifera*
MNCN_Ent 439785	A7	*Akis acuminata*	S Iberia	*Akis acuminata*
MNCN_Ent 439788	A7	*Akis acuminata*	S Iberia	*Akis acuminata*
MNCN_Ent 439773	A7	*Akis granulifera*	S Iberia	*Akis granulifera*
MNCN_Ent 439774	A7	*Akis granulifera*	S Iberia	*Akis granulifera*
MNCN_Ent 439775	A7	*Akis granulifera*	S Iberia	*Akis granulifera*
MNCN_Ent 439792	A7	*Akis acuminata*	N Morocco	*Akis acuminata*
MNCN_Ent 439804	A7	*Akis acuminata*	S Iberia	*Akis acuminata*
MNCN_Ent 439805	A7	*Akis acuminata*	S Iberia	*Akis acuminata*
MNCN_Ent 439789	A7	*Akis acuminata*	S Iberia	*Akis acuminata*
MNCN_Ent 439793	A7	*Akis acuminata*	N Morocco	*Akis acuminata*
MNCN_Ent 439794	A7	*Akis acuminata*	N Morocco	*Akis acuminata*
Tbr31	A7	*Akis acuminata*	S Iberia	*Akis acuminata*
MNCN_Ent 439795	A7	*Akis acuminata*	S Iberia	*Akis acuminata*
MNCN_Ent 439776	A7	*Akis granulifera*	S Iberia	*Akis granulifera*
MNCN_Ent 439800	A7	*Akis acuminata*	S Iberia	*Akis acuminata*
MNCN_Ent 439790	A7	*Akis acuminata*	S Iberia	*Akis acuminata*
MNCN_Ent 439803	A7	*Akis acuminata*	S Iberia	*Akis acuminata*
MNCN_Ent 439801	A7	*Akis acuminata*	S Iberia	*Akis acuminata*
MNCN_Ent 439802	A7	*Akis acuminata*	S Iberia	*Akis acuminata*
MNCN_Ent 439786	A7	*Akis acuminata*	S Iberia	*Akis acuminata*
MNCN_Ent 439796	A7	*Akis acuminata*	Central Iberia	*Akis acuminata*
MNCN_Ent 439787	A7	*Akis acuminata*	SE Iberia	*Akis acuminata*
MNCN_Ent 439791	A7	*Akis acuminata*	Central Iberia	*Akis acuminata*
MNCN_Ent 439797	A7	*Akis acuminata*	S Iberia	*Akis acuminata*
MNCN_Ent 439798	A7	*Akis acuminata*	S Iberia	*Akis acuminata*
MNCN_Ent 439799	A7	*Akis acuminata*	S Iberia	*Akis acuminata*
MNCN_Ent 439814	A8	*Akis discoidea*	SE Iberia	*Akis discoidea*
MNCN_Ent 439817	A8	*Akis discoidea*	SE Iberia	*Akis discoidea*
MNCN_Ent 439815	A8	*Akis discoidea*	SE Iberia	*Akis discoidea*
MNCN_Ent 439816	A8	*Akis discoidea*	SE Iberia	*Akis discoidea*
MNCN_Ent 439818	A8	*Akis discoidea*	SE Iberia	*Akis discoidea*

MNCN_Ent 439819	A8	*Akis discoidea*	SE Iberia	*Akis discoidea*
MNCN_Ent 439824	A8	*Akis discoidea*	SE Iberia	*Akis discoidea*
MNCN_Ent 439821	A8	*Akis discoidea*	SE Iberia	*Akis discoidea*
MNCN_Ent 439823	A8	*Akis discoidea*	SE Iberia	*Akis discoidea*
MNCN_Ent 439820	A8	*Akis discoidea*	SE Iberia	*Akis discoidea*
MNCN_Ent 439822	A8	*Akis discoidea*	SE Iberia	*Akis discoidea*
MNCN_Ent 439825	A8	*Akis discoidea*	SE Iberia	*Akis discoidea*
MNCN_Ent 439826	A9	*Akis tingitana*	Morocco	*Akis tingitana*
MNCN_Ent 439827	A9	*Akis tingitana*	Morocco	*Akis tingitana*
MNCN_Ent 439828	A10	*Akis goryi*	Tunisia	*Akis goryi*
MNCN_Ent 439829	A10	*Akis goryi*	Tunisia	*Akis goryi*
MNCN_Ent 439830	A11	*Akis trilineata*	Morocco	*Akis trilineata*
AKI8034	A12	*Akis bacarozzo*	Balearic Islands	*Akis bacarozzo*
MNCN_Ent 439831	A12	*Akis bacarozzo*	Balearic Islands	*Akis bacarozzo*
MNCN_Ent 439833	A12	*Akis bacarozzo*	Balearic Islands	*Akis bacarozzo*
MNCN_Ent 439834	A12	*Akis bacarozzo*	Balearic Islands	*Akis bacarozzo*
MNCN_Ent 439832	A12	*Akis bacarozzo*	Balearic Islands	*Akis bacarozzo*
MNCN_Ent 439835	A12	*Akis bacarozzo*	Balearic Islands	*Akis bacarozzo*
MNCN_Ent 439836	A12	*Akis bacarozzo*	Balearic Islands	*Akis bacarozzo*
MNCN_Ent 439837	A13	*Akis heydeni*	Morocco	*Akis heydeni*
MNCN_Ent 464948	M1	*Morica planata*	S Iberia	*Morica planata*
MNCN_Ent 464951	M1	*Morica planata*	S Iberia	*Morica planata*
MNCN_Ent 464949	M1	*Morica planata*	N Morocco	*Morica planata*
MNCN_Ent 464952	M1	*Morica planata*	Central Morocco	*Morica planata*
MNCN_Ent 464953	M1	*Morica planata*	Central Morocco	*Morica planata*
MNCN_Ent 464950	M1	*Morica planata*	S Morocco	*Morica planata*
AKI8031	M1	*Morica planata*	N Morocco	*Morica planata*
MNCN_Ent 464954	M2	*Morica favieri*	SE Iberia	*Morica favieri*
MNCN_Ent 464955	M2	*Morica favieri*	SW Morocco	*Morica favieri*
MNCN_Ent 464956	M3	*Morica hybrida*	SE Iberia	*Morica hybrida*
