# Supplementary material for: Morphological and Cyto-Nuclear Conflicting Signals Across Non-Sister Lineages in Darkling Beetles (Tenebrionidae: Akis)
Source: Genes (Basel). 2026 Apr 14;17(4):455. doi: 10.3390/genes17040455 (PMC13116412; doi:10.3390/genes17040455)
Supplement: Supplementary file 1 [file genes-17-00455-s001.zip › Table_S2_supplementary_file.pdf]

Table S2. Main morphological diagnostic traits of the species of *Morica* and *Akis*. Sources: [58-61, 81, 95-97], and pers. obs. (authors).

	Body size	Pronotum shape	Pronotal margins: shape	Pronotal margins: appearance	Pronotum hind angles (♂)	Pronotum disc	Elytral capsule shape (dorsal view)	Elytral disc	Elytral surface	Elytral ribs: number and situation	Elytral ribs: appearence	Appearance of elytral integument	Aedeagus
Morica planata	Large-medium (21-30 mm)	Width, transverse	Not elevated, moderately narrow	Almost smooth, with few faint transverse wrinkles	Obtuse, not protruding	Moderately convex	Ovoid, hind rounded	Convex	Smooth	1 marginal, 1 incomplete humeral, 1 very weak or absent dorso-lateral	Moderately fine, toothed or semi-toothed	Satin or semi-glossy	Moderately robust
Morica hybrida	Medium (15-22 mm)	Width, transverse	Not elevated, moderately narrow	With wide and diffuse transverse wrinkles	Obtuse, not protruding	Moderately convex	Ovoid, hind rounded	Convex	Markedly rough-reticulated	1 entire marginal, 1 incomplete humeral, 2 incomplete dorsal	Fine and smooth	Matte, opaque	Moderately robust
Morica favieri	Large-medium (17-25 mm)	Width, transverse	Not elevated, moderately narrow	With wide transverse wrinkles	Obtuse, not protruding	Moderately convex	Ovoid, hind rounded	Convex	Faintly rough, with 3 or 4 incomplete series of medium pointed tubercles	1 entire marginal	Moderately fine, toothed	Semi-glossy	Moderately robust
Akis discoidea	Medium (15-23 mm)	Moderately narrow	Elevated, relatively wide	Almost smooth, with few faint transverse wrinkles	Acute and protruding	Flat	Fusiform, weakly or moderately widened	Convex	Smooth	Lacking ribs or with a very weak dorsal and/or humeral fold	Smooth	Semi-matte	Slender
Akis acuminata	Medium (16-24 mm)	Moderately narrow	Elevated, relatively wide	With faint and diffuse transverse wrinkles	Acute and protruding	Flat	Fusiform, widened	Weakly convex	Smooth	1 entire marginal, sometime 1 incomplete humeral	Fine, smooth or weakly toothed	Satin or semi-glossy	Slender
Akis elegans	Medium (18-23 mm)	Moderately narrow	Elevated, moderately narrow	Smooth, lacking or weakly transverse wrinkles	Acute and protruding	Flat	Subfusiform, barely widened in the middle	Flat between the dorsal ribs	Smooth	1 marginal, 1 humeral (close to the marginal in its anterior half), 1 mid-dorsal	High, finely keeled and subtoothed	Matte, opaque	Slender
Akis bacarozzo	Medium (16-20 mm)	Moderately wide	Elevated, moderately wide	With strong transverse wrinkles	Acute and protruding	Weakly convex	Subfusiform, moderately widened in the middle	Flat or very slightly convex	Smooth, with a lateral series of large pointed tubercles	1 marginal	Fine, toothed	Semi-glossy	Slender
Akis tingitana	Medium (16-21 mm)	Moderately wide	Weakly elevated, moderately narrow	With small, faint transverse wrinkles	Acute and protruding	Flat	Subfusiform, moderately widened in the middle	Flat	Microgranulated, with three series of medium or large pointed tubercles	1 marginal	Fine, barely toothed	Semi-matte or satin	Slender
Akis goryi	Large-medium (19-26 mm)	Moderately wide, transverse	Weakly elevated, wide	Almost smooth or with few, weak wrinkles	Acute and protruding	Flat	Subfusiform, barely widened in the middle	Flat between the dorsal ribs	Smooth	1 marginal, 1 mid-dorsal	High, finely keeled and toothed	Semi-matte or satin	Slender
Akis trilineata	Large-medium (18-26 mm)	Moderately narrow, not transverse	Moderately elevated, relatively narrow	With sharp transverse wrinkles	Acute and protruding	Flat or weakly convex	Fusiform, weakly widened in the middle	Weakly convex	Smooth	1 marginal, 1 incomplete humeral, 1 weak, incomplete dorso-lateral	Moderately fine, toothed and/or crenulated	Satin or semi-glossy	Slender
Akis heydeni	Large-medium (18-28 mm)	Moderately narrow, not transverse	Moderately elevated, relatively narrow	With faint and diffuse transverse wrinkles	Right or slightly acute, weakly protruding	Flat	Fusiform, weakly widened in the middle	Flat between the dorsal ribs	Smooth	1 marginal, 1 humeral, 1 (very variable) mid-dorsal	Moderately elevated, finely keeled and crenulated	Satin or semi.glossy	Slender
Akis lusitanica	Medium (16-22 mm)	Moderately wide, transverse	Weakly elevated, wide	Almost smooth or with few, weak transverse wrinkles	Acute and protruding	Flat	Fusiform, moderately widened in the middle	Flat between the dorsal ribs	Smooth	1 marginal, 1 humeral, 1 weak, incomplete dorso-lateral	Slightly elevated, moderately fine and weakly crenulated	Semi-matte or satin	Slender
Akis granulifera	Large-medium (19-25 mm)	Moderately wide, transverse	Moderately elevated, wide	With deep transverse wrinkles	Acute and protruding	Flat or weakly convex	Fusiform, markedly widened in the middle	Weakly convex between the dorsal ribs	Smooth, with or without series of sparse tubercles between the ribs	1 marginal, 1 humeral, 1 dorso-lateral	Highly elevated, moderately thick and toothed	Glossy	Slender
Akis genei	Medium (17-23 mm)	Moderately wide, transverse	Moderately elevated, wide	With scarce, weak and difusse transverse wrinkles	Acute and protruding	Flat or weakly convex	Fusiform, moderately widened in the middle	Weakly convex between the dorsal ribs	Densely microgranulated	1 marginal, 1 humeral, 1 marked or weak, incomplete dorso-lateral	Sligthly or moderately elevated, fine and weakly crenulated	Satin or semi-glossy	Slender
